# Acupuncture on the Endometrial Morphology, the Serum Estradiol and Progesterone Levels, and the Expression of Endometrial Leukaemia-inhibitor Factor and Osteopontin in Rats

**DOI:** 10.1155/2011/606514

**Published:** 2010-09-30

**Authors:** Houju Fu, Yuanqiao He, Ying Gao, Yicun Man, Wukun Liu, Hua Hao

**Affiliations:** ^1^Department of Obstetrics and Gynecology, Union Hospital, Tongji Medical College, Huazhong University of Science and Technology, Wuhan Hubei 430022, China; ^2^Department of Urology, Union Hospital, Tongji Medical College, Huazhong University of Science and Technology, Wuhan Hubei 430022, China; ^3^Department of Gynecology, Endometriosis Research Center, Charité, Campus Benjamin Franklin, Hindenburgdamm 30, 12200 Berlin, Germany; ^4^Institute of Pharmacy, Free University of Berlin, Koenigin Luise Strasse 2+4, 14195 Berlin, Germany; ^5^Department of Pathophysiology, Tongji Medical College, Huazhong University of Science and Technology, Wuhan Hubei 430022, China

## Abstract

Although it is well known that acupuncture has beneficial effects on a variety of medical conditions especially in pain relief, nausea, and vomiting, it remains controversial whether it has positive impact on the female reproduction. The present study aimed to evaluate whether the following endometrial receptivity factors: the endometrial morphology, the hormone concentrations, and the protein expression of endometrial leukaemia-inhibitory factor (LIF) and osteopontin (OPN) could be improved by the acupuncture in clomiphene citrate(CC)-induced rat model during implantation period. Results showed that, compared with the CC group, glandular development advanced, the serum estradiol levels decreased significantly, and the glandular area and endometrial LIF and OPN expression were significantly higher in acupuncture group. There were no significant differences in serum progesterone levels, endometrial thickness, and stromal area between groups. These results suggest that acupuncture can improve certain aspects of endometrial receptivity in CC-induced rat model during implantation period, which might result in endometrial state better to female reproduction.

## 1. Introduction

Acupuncture (AC), one of the key components of Traditional Chinese Medicine (TCM), is used at certain points on the body that relate to special pathways known as meridians to mobilize the Qi (Chee) stuck, based on the notion that Qi, the vital energy of body health, circulates and moves freely along meridians to maintain the body in a ‘balanced state' between “Yin” and “Yang” [1]. This therapy is a safe and effective holistic medicine backed by more than three thousand years of practice and research and has been gaining increasing popularity in the west world as complementary and alternative therapeutic intervention [2].

AC has been used for a variety of medical conditions in both adults [2, 3] and children [4]: pain relief, nausea and vomiting, drug addiction, stroke rehabilitation, depression, irritable bowel syndrome, and asthma, to name a few. A National Institutes of Health consensus development panel released a survey of AC, indicating that it has beneficial effects for surgery and chemotherapy-related nausea and vomiting and pain [5].

Recently, AC has been administrated in assisted reproductive technology to enhance the success of in vitro fertilization (IVF) treatment, due to the relatively low success rate of IVF per cycle, as well as the high emotional and financial costs. Some investigations have already demonstrated that AC had positive effects in anovulatory subfertility [6, 7], male subfertility [3, 8], pain relief during oocyte retrieval [2, 9, 10], IVF implantation rates [11, 12], and the quality of life of patients undergoing IVF [2]. Also, there are reviews suggesting that certain effects of AC are needed by uterus [13] and it improves pregnancy rates and live birth among women undergoing IVF [14]. However, the definitive role of AC in infertility is still unclear and controversial [2, 3]. 

AC has specific Qi-Xue mechanism, channels, De-qi therapy, and manipulation, which are completely different from the ordinary drug administration. Its mechanism remains a mystery under the condition of current scientific technologies. Even, its placebo assessment has become a subject of controversy [15–18]. More clinical trials and basal researches that are consistent with TCM principles are required to provide a greater understanding for the clinical applicability of AC. 

Clomiphene citrate (CC), a nonsteroidal compound, is one of the remarkably common drugs used in infertile women especially those with polycystic ovary syndrome, and is thought to have oestrogenic and antioestrogenic properties. Its antiestrogenic effect in hypothalamic and pituitary is exactly what we need, through inhibiting estrogen receptors to result in a favorable alteration in the characteristics of pulsatile gonadotropin-releasing hormone secretion and then the initiation of ovulation [19], while its interacting with other tissues such as ovary and endometrium may lead to underlying pathophysiologic negative influence on reproductive tract [20–22], which induce discrepancy between ovulation rate (60%–85%) and conception rate (20%) [19].

The aim of this study was to investigate whether AC administration would improve these side effects of CC on the endometrium histology, endometrium receptivity cytokine, and the serum estradiol(E2) and progesterone(P) in CC-induced rat model and to provide evidence for the use of AC in reproduction.

## 2. Materials and Methods

### 2.1. Animals

Sexually mature female Sprague-Dawley rats, weighing 200–230 g were caged in animal colony according to the institutional guidelines established by the Animal Care and Use Committee of Tongji Medical College, Huazhong University of Science and Technology. The rats were fed with a rodent diet produced by school animal center and water freely, under the controlled temperature (25 ± 0.5°C) and a 12-hour light-dark regimen (lights on from 7:00 AM to 7:00 PM). The rodent diet is mainly made of corn, flour, dehydrated soybean meal, extruded soybean meal, fish meal, wheat bran, and alfalfa, according to the national standards, which do not affect hormonal level. The rats were housed for at least one week of acclimatization period before the experiment.

### 2.2. Tissue Preparation

Estrus was identified by daily vaginal smear. Only rats that had exhibited estrus for more than two consecutive periods of regular 4-day cycles were recruited into the present study. Suitable rats were randomly allocated to three groups, each including 11 subjects.

Rats in CC group were administered with Clomifene Citrate Tablets (Chinese name: Fa Di Lan. Codal Synto Limited, Cyprus) 100 mg/kg/day dissolved in 2 mL of tap water via lavage at 9.00 AM-11.00 AM for 2 days, beginning from the 1st day of diestrus stage of estrous cycle. On day 3, they were mated after being given the intraperitoneal injection of human chorionic gonadotrophin (Livzon Pharmaceutical Group Inc., Guangdong, China) at 0.1 IU/g bw at 5.00 p.m. If sperms were observed in the vaginal smear specimens on the 4th day morning, we regarded that the rats were pregnant, and defined day 4 as the 1st day of pregnancy. In AC group, besides the procedures mentioned above, conscious rats which were swung in a self-made linen sack and kept in standing (prone) position were administrated with AC for 25 minutes in the afternoon once a day for 6 days, from the same day of CC administration to the 3rd day of pregnancy. The normal control (NC) group was composed of untreated pregnant rats, which were naturally mated during their estrus phases.

The three groups of rats were anesthetized with 10% Chloral Hydrate at noon on the 5th day of pregnancy. Bloods were drawn through cardiac puncture for measurement of serum E2 and P, and the sera were separated and stored at −20°C until later assay. Then rats were sacrificed by cervical dislocation and the whole uteri were collected promptly, with the excess fat and connective tissue trimmered off. Left uterine horns were transected and fixed in 10% formalin for immunohistochemical study while the right samples including both endometrium and myometrium were stored at −80°C until the extraction of protein. The study design was shown in Table 1.

### 2.3. AC Treatment

The 16 mm long sterile disposable filiform needles (Shanghai Taicheng Technology Development Co., Ltd, Shanghai, China) were inserted to depths of 1–7 mm, then lifted, and twirled by hand evenly every 5 minutes. The stimulated points were as follows: bilateral Sanyinjiao (SP6), bilateral Zusanli (ST36), bilateral Taichong (LR3), Guanyuan (CV4), and Zhongji (CV3). The depth and the location of the points were based on the concepts of TCM, the atlas of skeleton and acupoints of rat [23], and the anatomical location described in previous research [24]. Sanyinjiao (SP6) is situated approximately 10mm directly above the tip of the medial malleolus, on the posterior border of the tibia. Zusanli (ST36) is located 4-5 mm lateral to the anterior tubercle of the tibia, posterolaterally to the distal end of the cranial tuberosity of the tibia, in the tibialis anterior muscle, innervated by the deep peroneal nerve. Taichong (LR3) is on the dorsum of the hind limb, in the depression distal to the junction of the first and second metatarsal bones. Zhongji (CV3) is located at the point of 4/5 down the ventral midline connecting the umbilicus to the pubic tubercle. Guanyuan (CV4) is located at the point of 3/5 down the ventral midline connecting the umbilicus to the pubic tubercle.

### 2.4. Radioimmunoassay (RIA)

Sera E2 and P were analyzed by RIA kits (S10940094, Beijing North Institute of Biological Technology, Beijing, China) in the Department of Nuclear Medicine of Tongji Hospital. All sera were assayed on the same day to avoid interassay variation. The samples were analyzed in serial dilutions optimized to linear part of the standard curve and corrected for nonspecifc binding. The intra-assay coeffcient of variations of both E2 and P were less than 10%. For E2, the detection limit was less than 2 pg/mL. The rates of cross reactivity of E2 with estriol, *P* and testosterone were 0.016%, 0.01% and 0.01%, respectively. The detection limit of *P* was 2 ng/mL and the reference intervals were 0.2–100 ng/mL.

### 2.5. Hematoxylin and Eosin Staining

After fixed with 10% formalin for 24 hours and embedded in paraffin, the uteri were cut into a thick 5 *μ*m transverse sections and then mounted on slides. Parts of these tissue sections were stained with hematoxylin (Harris) and eosin according to the standard procedure. Morphological changes were observed under light microscope, and morphometric parameters were evaluated by high-resolution medical color image automatic analysis system (HMIAS)-2000 (Wuhan Champion Image Technology Co., Ltd, China).

### 2.6. Immunohistochemistry

Other parts of sections were also stained using standard procedures of immunohisto- chemistry staining. Deparaffinized in xylene and dehydrated in a series of ethanol solutions, they were then covered in 0.3% hydrogen peroxide for 20 minutes to block endogenous peroxidases. Antigen retrieval was performed by incubating the sections in 10 mmol/L citrate buffer solution (pH 6.0) at 100°C for 5 minutes and cooling naturally at room temperature. The sections were incubated with leukaemia inhibitory factor (LIF) purified goat polyclonal antibody (SC-1336, Santa Cruz, CA, USA) or osteopontin(OPN) mouse-anti-rat monoclonal antibody (SC-21742, Santa) diluted 1 : 100 overnight at 4°C, respectively. After washing in PBS, the sections were incubated with biotinylated goat antimouse IgG or rabbit antigoat IgG, followed by SABC Solution(Boster Biological Technology, Ltd. Wuhan, China) for 30 minutes at room temperature and diaminobenzidine (DAB Kit, Boster Biological Technology, Ltd. Wuhan, China) for about 10 min, during which the brown colour was controlled under light microscope. Staining intensity of tissue sections was also evaluated by HMIAS-2000. The deeper the color shows, the greater the value of optical density becomes.

### 2.7. Western Blot

Rat endometrium tissues were homogenated and lysed in extraction buffer (50 mmol/L Tris-HCl (pH 7.4), 150 mmol/L NaCl, 1% NP-40, 0.5% sodium deoxycholic acid, 0.1% SDS, 100 *μ*g/mL PMSF, and 100 *μ*g/mL leupeptin). The protein concentration was quantified using the BCA protein assay kit (Prod#23227, Pierce, IL, USA). After denatured through incubating at aqua bulliens for 5min, the protein was electrophoresed with sodium dodecyl sulphate-polyacrylamide gel electrophoresis (SDS-PAGE) using 10% polyacrylamide gels and transferred to nitrocellulose membranes (1620115, Bio-Rad, Hercules, CA, USA). The membranes were blocked with 5% fat-free powdered milk in TBS-T (10 mmol/L Tris, 150 mmol/L NaCl, and 0.05% Tween-20, pH 8.0) at room temperature for 1 hour and incubated overnight in 1% TBS-T at 4°C with LIF purified goat polyclonal antibody (SC-1336, Santa) or OPN mouse-anti-rat monoclonal antibody (SC-21742, Santa) diluted 1 : 1000. Following three washes with TBS-T, blots were incubated with the appropriate secondary antibody IgG-horseradish peroxidase conjugate at dilution of 1 : 2000 at room temperature for 1 hour. After final washing with TBST, the membranes were detected by enhanced chemiluminescence (Prod#34079, Pierce) and exposed to X-ray films. Expression of target proteins was internally normalized to *α*-Tubulin (SAB3500023, Sigma, USA).

### 2.8. Statistical Analysis

Data were analyzed by SPSS 12.0 statistical software (SPSS Inc., Chicago, USA). The Kruskal-Wallis test was used to detect overall differences among three groups, followed by Mann-Whitney U-test for multiple comparisons. Results are expressed as mean ± SD. *P* value <.05 was considered significant in 2 × 2 comparisons.

## 3. Results

### 3.1. Endometrial Morphology

**Figure 1:**
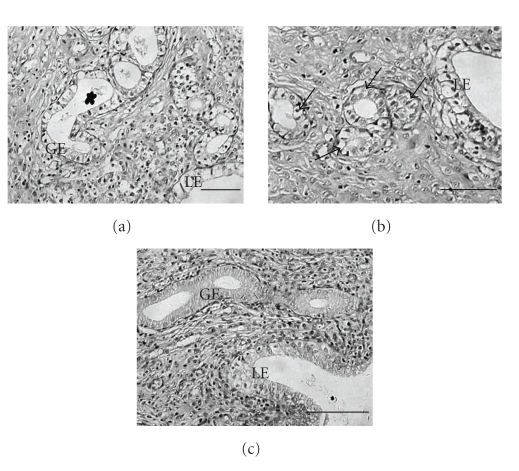
Comparison of endometrial morphology during implantation period in rats. Specimens from NC group (a) showed normal endometrium: well-arranged luminal epithelium (LE), abundant glandular secretions (asterisk in (a)) and coherent subnuclear vacuoles in both LE and glandular epithelium (GE). Endometrial tissues of intervention groups showed glandular-stromal dyssynchrony: no secretions but stroma similar to normal. Subnuclear vacuoles of CC group (c) were only shown in LE, but they appeared in both LE and GE after acupuncture treatment (arrows in (b)). Original magnification: ×200. Scale bar** =** 100 *μ*m.

Only the endometrial layer was subjected to analysis, and the same outcome was obtained by two independent observers blind to treatment. Morphological changes were shown in Figure 1. Endometrial tissues from pregnant rats in NC group (Figure 1(a)) showed well-arranged luminal epithelium (LE), abundant glandular secretion and coherent subnuclear vacuoles in almost all LE and glandular epithelium (GE). There was no secretion in AC (Figure 1(b)) and CC groups (Figure 1(c)), and stromal morphology did not significantly differ among three groups. However, subnuclear vacuoles of AC group appeared in GE as well as in LE when compared with CC group.

Morphometric parameters and data of endometria detected were shown in Table 2. Glandular area differed significantly among the three groups (all *P* < .05). Endometrial thickness and stromal area were substantially different in the NC group than in the CC group (both *P* < .01), but not in the AC group than in the CC group (both *P* > .05).

### 3.2. E2 and P Concentrations in RIA

Table 3 summarized the serum hormone levels in the three groups during implantation period in rat model. The serum E2 concentration of the CC group was higher than that of the NC group (*P* < .01), and the comparison of that between the AC and CC groups showed a significant decrease (*P* < .05). However, the serum P concentration did not significantly differ among the three groups in our present trial (*P* > .05).

### 3.3. Expression of Endometrial LIF and OPN Proteins in Immunohistochemistry

**Figure 2:**
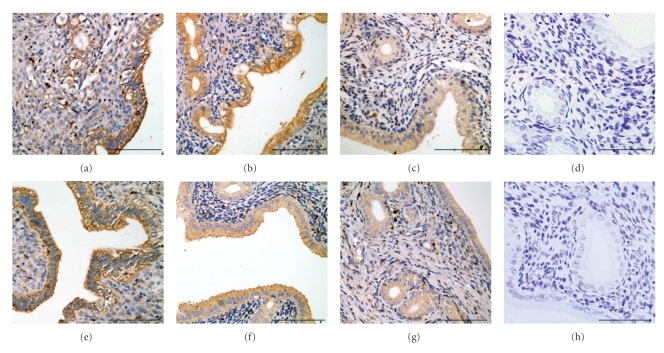
Immunohistochemical staining for the expression of endometrial leukaemia inhibitory factor (LIF) and osteopontin (OPN) during implantation period in rat. Note that immunostaining of the LIF ((a)–(d)) and OPN ((e)–(h)) was mainly detected on glandular and luminal epithelial cells. Staining intensity of both LIF and OPN in AC group was significantly weaker than that of NC group but stronger compared with CC group. When using PBS instead of the anti-LIF antibody (d) or the anti-OPN antibody (h), no positive signal was observed. ((a) and (e): NC group; (b) and (f): AC group; (c) and (g): CC group). Original magnification: ×200. Scale bar = 100 *μ*m.

**Figure 3:**
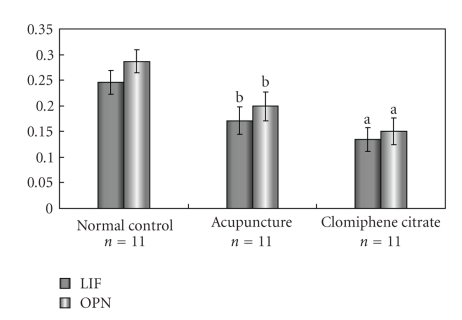
The immunohistochemical staining intensities of leukaemia-inhibitory factor (LIF) and Osteopontin (OPN) proteins in rat endometria during implantation period. Data are expressed as mean ± SE. (a), *P* < .01 compared with Normal control group; (b), *P* < .01 compared with Clomiphene citrate group.

The immunostaining for LIF (Figures 2(a)–2(d)) and OPN (Figures 2(e)–2(h)) was predominantly detected in GE and LE in rat endometrium, with a little expression in stroma. Staining intensity of LIF in AC group was weaker than that of NC group (optical density: 0.171 ± 0.027 versus 0.246 ± 0.023), but higher than that of CC group (optical density: 0.135 ± 0.023).The same trend of immunoreactivity of OPN was observed in the three groups. (optical density: 0.287 ± 0.022 in NC group, 0.199 ± 0.028 in AC group, and 0.150 ± 0.026 in CC group). All the comparisons above had significant differences (*P* < .01) and the staining intensities are depicted in Figure 3.

### 3.4. Expression of Endometrial LIF and OPN Proteins in Western Blot

**Figure 4:**
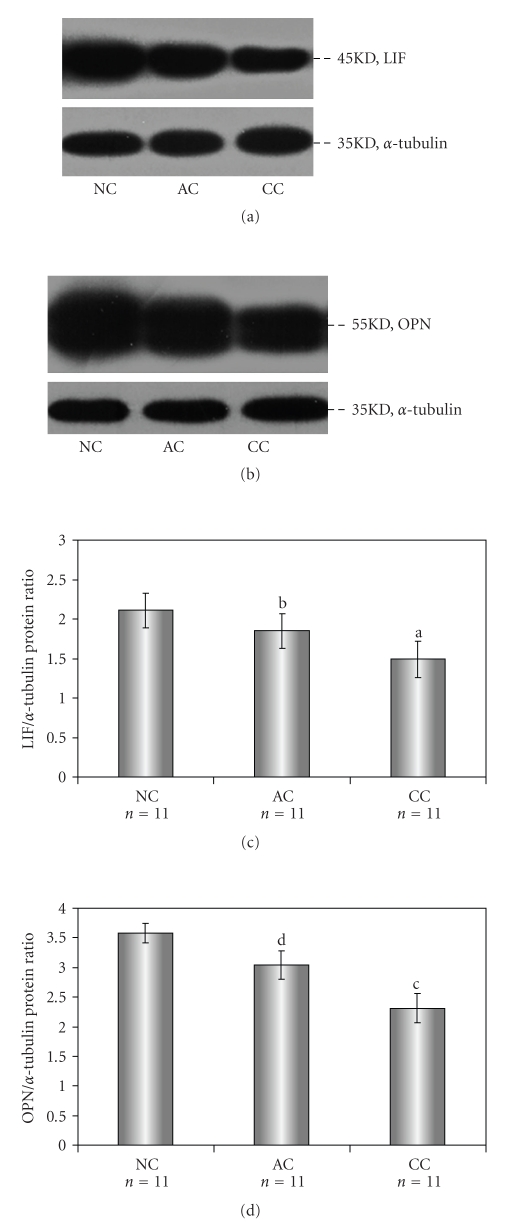
Expression of endometrial leukaemia-inhibitory factor (LIF) and osteopontin (OPN) proteins during implantation period in rats ((a) and (b)). Expression of target proteins was internally normalized to the optical density of *α*-Tubulin ((c) and (d)). Data are expressed as mean ± SE of the LIF/*α*-Tubulin protein ratio and OPN/*α*-Tubulin protein ratio: (a) and (c), *P* < .01 compared with NC group; (b) and (d), *P* < .01 compared with CC group.

Consistent with the results of immunohistochemical staining, the expression trends of LIF and OPN proteins were confirmed by Western blot analyses (Figure 4(a) and 4(b)). The band of *α*-Tubulin was used as an internal loading control in each lane. Normalized with *α*-Tubulin expression level (Figures 4(c) and 4(d)), the expression of both LIF and OPN proteins in AC group were lower than those in the NC group, but higher as compared with CC group: *P*-values of <.01 in CC group versus NC group and *P*-values of <.01 in AC group versus CC group.

## 4. Discussion

**Figure 5:**
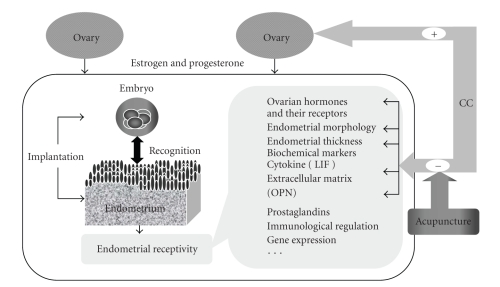
Under the influence of ovarian hormones, endometrial receptivity that is, rigorously controlled both temporally and spatially embraces various factors. Some of them, such as endometrial morphology, biochemical markers-leukaemia inhibitory factor (LIF), and osteopontin (OPN), as well as ovarian hormones are negatively influenced by clomiphene citrate (CC), which were improved by acupuncture in present trial. (+): The effect of CC on ovulation. (−): The adverse effects of CC on reproduction.

Under the present high dose in our experiment, the rat model with high-serum E2, thin and impaired endometrium confirmed the side effects of CC [20–22] and resulted in abnormal endometrial receptivity which may cause the failure of approximately two-thirds of implantations [25]. The process of implantation, classically classified into three stages: apposition, adhesion, and invasion, involves a complex sequence of recognition signaling events between the synchronous stage conceptus and the primed and receptive uterine. It only takes place during the limited “implantation window”, a restricted period of endometrial receptivity spanning, between days 20 and 24 of a regular menstrual cycle in humans [26, 27], days 4 and 6 of pregnancy in rats [28, 29]. Endometrial receptivity that is rigorously controlled both temporally and spatially embraces various factors under the influence of ovarian hormones, including morphological features, cellular adhesion molecules family, cytokines, prostaglandins, and immunological regulation [26, 27], as is shown in Figure 5. 

Endometrial morphology can be accomplished by histological dating of the endometrial biopsy specimen serving as an important bioassay. Not only there is evidence suggesting that sampling during the implantation window is quite sensitive for identifying endometrial maturation [30], but some arguers, holding that the morphological change can't be used to assess the reproductive ability, also thought that the abnormal secretary endometrial maturation may adversely affect reproductive performance [31]. Previous researches found that AC administration had positive effect on morphology, such as the ultrastructural integrity of spermatozoa in human [32] and matured follicles rupture and conversion into corpus luteum in mice [33]. A pilot study has displayed that the combination of AC and Sildenafil suppositories increased endometrial thickness by upregulating nitric oxide synthase [34]. In terms of our data in present study, not the endometrial thickness (*P* = .07) but the glandular area (*P* = .01) of endometrium was significantly increased in the AC group compared with the CC group. So we suggested that AC may have capacity to mainly stimulate the growth of gland rather than stoma, which cannot be observed by ultrasound in clinics.

Sex steroids as systemic factors regulate the uterine receptivity and implantation process as well as menstruation through their ligand receptors expressed in the epithelial, stromal, and vascular cells. Estrogen enhances endometrial cell proliferation, and progesterone leads to the differentiation of these cells following ovulation. The latter hormone is required for implantation and pregnancy maintenance in all mammals, although the requirement for estrogen is species-specific [35]. In clinic, as we know, high E2 concentration will lead to ovary hyperstimulation syndrome. At present study, AC significantly suppressed the high-serum E2 induced by CC to bring better implantation state. Preliminary studies have also found that AC was effective with the management of sex hormones. It could adjust serum follicle stimulating hormone (FSH), luteinizing hormone, and E2 in women suffering from ovulatory dysfunction [7], and in rats with primary dysmenorrheal [36]. Recently, in a randomized controlled trial, AC and auricular treatment significantly decreased the FSH level while increased the E2 level to relieve the menopausal hot flashes of the bilaterally ovariectomized women [37]. The serum concentrations of P in our study were similar among the three groups, which did not support the result described before [38], but we observed that the serum *P* concentrations in six out of eleven samples of CC group were much lower than the normal level (<1 ng/mL), and the same phenomenon of CC-induced low P concentration has also been found in other studies [39, 40].

The present study showed that AC improved the expression of LIF and OPN proteins in a rat's uterus during implantation period. It must be closer to normal state. The two cellular factors largely accepted as the promising candidates of biomarkers of endometrium receptivity are critical to the establishment of blastocyst implantation and pregnancy. The important role for LIF was obviously shown on knockout mice's failure to implant, whereas LIF (-/-) blastocysts can be successfully transplanted into wild-type recipient females [41]. OPN acts as an adhesive, like a bridge between its ligand integrin *α*v*β*3 on the embryo and epithelium [42]. OPN mRNA was highly expressed in human endometrium during periimplantation period or implantation window in several gene that profiling researches [43–45]. It has been demonstrated that AC stimulation affected cytokine production. Not only did it modulate immune Th1 and Th2 cytokines in the hypothalami of rats with lipopolysaccharide-induced fever [46] and in the ventral midbrains of healthy rats [47], but also increased the release of *β*-endorphin in the brain [48]. Besides, electro-AC has been found to raise the neuropeptide Y concentrations in follicular fluid [49] and reduce uterine motility in pregnant rats by inhibiting the cyclooxygenase-2 expression of the endometrium [50].

The biological mechanisms of AC's impact on reproduction are still unclear. It has been established, however, that the positive effect of AC in the treatment of infertility is related to the following [2, 3]: (a) modulating the release of neurotransmitters, such as *β*-endorphin, which then influence the hypothalamic functions (b) increasing uterine and ovarian blood flow to improve endometrial environment (c) inhibiting uterine contraction to reduce the possibility of expelling embryo out of the uterus (d) modulating immune factors (e) reducing stress of subfertile patients. The acupoints chosen in the present trial have been found to be associated with regulating gonadal hormone levels in both human [7, 37] and animal [36, 51–63] and improving pregnancy rate of IVF [11, 12, 14, 54, 55]. According to the TCM principles, Sanyinjiao (SP6) is considered as a classic acupoint for female disorders and can soften and harmonize the liver and benefit the kidney Qi [56, 57]. Zusanli (ST36) is the main tonification acupoint for strengthening the body's resistance and restoring vital energy, and Taichong (LR3) spreads the stagnation of liver Qi and nourishes liver blood [57]. Both Zhongji (CV3) and Guanyuan (CV4) are on the Conception Vessel, which can nourish the uterus to adjust the axis function [7, 57]. The days of AC administration were also in the light of previous studies in rat [52, 53]. Recently, acupoint specificity [58–61] and sham needling [15–18] of AC have been subjects of controversy, but there are studies that link particular acupoints with unique connective tissue location [62], the meridian with unique electrical property [63].In the experiment, we just use the normal pregnant rats as the control, because ‘sham' designs study a simulacrum of AC, and a high-placebo model should be found [64]. 

In conclusion, AC was found in our study to ameliorate the uterine environment, by advancing the gland development, reducing the high-serum E2 concentration, and increasing the glandular area and the expression of receptivity markers: LIF and OPN proteins during the implantation period in CC-induced model. It may be a valuable complementary and alternative treatment for female reproduction. However, further rigorous research is needed to confirm this physiologic effectiveness of AC.

## Figures and Tables

**Table 1:** The design of this study.

Day/group	Group CC	Group AC	Group NC	Estrus cycle	Duration of pregnancy
Day 1	CC (100 mg/kg)	CC (100 mg/kg) acupuncture		Diestrus	
Day 2	CC (100 mg/kg)	CC (100 mg/kg) acupuncture		Diestrus proestrus	
Day 3	HCG (0.1 IU/g) mated	Acupuncture HCG (0.1 IU/g) mated	Mated	Proestrus estrus	
Day 4	Found sperm	Found sperm acupuncture	Found sperm		The 1st day
Day 5		Acupuncture			The 2nd day
Day 6		Acupuncture			The 3rd day
Day 7					The 4th day
Day 8	Drew blood collected uterus	Drew blood collected uterus	Drew blood collected uterus		The 5th day

CC: clomiphene citrate; AC: acupuncture; NC: normal control; HCG: human chorionic gonadotrophin.

**Table 2:** Comparison of the morphometric characteristics of endometria in rats during implantation period.

Characteristics	Normal control	Acupuncture	Clomiphene citrate	*P* value	
	*n* = 11	*n* = 11	*n* = 11		
Thickness (*μ*m)	*621.25 ± 16*	441.31 ± 24	422.90 ± 17	.00^a^	.07^b^
Glandular area (mm^2^)	0.21 ± 0.03	0.17 ± 0.03	0.13 ± 0.03	.00^a^	.01^b^
Stromal area (mm^2^)	2.50 ± 0.35	1.80 ± 0.36	1.76 ± 0.36	.00^a^	.65^b^

^a^Normal control group versus clomiphene citrate group.

^b^Acupuncture group versus clomiphene citrate group.

**Table 3:** Comparison of the serum oestradiol and progesterone concentrations during implantation period in rat model.

Level	Normal control	Acupuncture	Clomiphene citrate	*P* value	
	*n* = 11	*n* = 11	*n* = 11		
Estradiol (pg/mL)	1472.00 ± 657	1899.73 ± 730	2617.63 ± 795	.00^a^	.03^b^
Progesterone (ng/mL)	2.03 ± 0.45	1.53 ± 0.60	1.47 ± 0.96	.12^a^	.70^b^

^a^Normal control group versus clomiphene citrate group.

^b^Acupuncture group versus clomiphene citrate group.
